# Screening for a Potential Therapeutic Agent from the Herbal Formula in the 4^th^ Edition of the Chinese National Guidelines for the Initial-Stage Management of COVID-19 via Molecular Docking

**DOI:** 10.1155/2020/3219840

**Published:** 2020-12-22

**Authors:** Yue Sun, Angela Wei Hong Yang, Andrew Hung, George Binh Lenon

**Affiliations:** ^1^School of Health and Biomedical Sciences, RMIT University, Melbourne 3083, Australia; ^2^School of Science, RMIT University, Melbourne 3083, Australia

## Abstract

**Background:**

COVID-19 caused by SARS-CoV-2 infection has been spreading through many countries since the end of 2019. The 4^th^ edition of the national guidelines for the management of COVID-19 provides an herbal formula with 9 herbs for its management. *Aim of Study*. We aimed to predict the mechanism of binding of SARS-CoV-2 and SARS-CoV spike glycoproteins with angiotensin-converting enzyme 2 (ACE2) to provide a molecular-level explanation of the higher pathogenicity of SARS-CoV-2 and to identify protein sites which may be targeted by therapeutic agents to disrupt virus-host interactions. Subsequently, we aimed to investigate the formula for the initial-stage management to identify a therapeutic agent with the most likely potential to become pharmaceutical candidate for the management of this disease.

**Materials and Methods:**

GenBank and SWISS-MODEL were applied for model creation. ClusPro was used for protein-protein docking. PDBePISA was applied for identification of possible binding sites. TCMSP was employed for identification of the chemical compounds. AutoDock Vina together with PyRx was used for the prediction and evaluation of binding pose and affinity to ACE2. SwissADME and PreADME were applied to screening and prediction of the pharmacokinetic properties of the identified chemical compounds. PyMOL was used to visualise the structural models of SARS-CoV-2 and SARS-CoV spike glycoproteins complexed to ACE2 and to examine their interactions.

**Results:**

SARS-CoV-2 had two chains (labelled chains B and C) which were predicted to bind with ACE2. In comparison, the SARS-CoV had only one chain (labelled chain C) predicted to bind with ACE2. The spike glycoproteins of both viruses were predicted to bind with ACE2 via position 487. Molecular docking screening and pharmacokinetic property prediction of the herbal compounds indicated that atractylenolide III (−9.1 kcal/mol) from *Atractylodes lancea (Thunb.) Dc.* (Cangzhu) may be a candidate therapeutic agent for initial-stage management.

**Conclusions:**

Atractylenolide III is predicted to have a strong binding affinity with ACE2 and eligible pharmacokinetic properties, anti-inflammatory effects and antiviral effects in *in vitro* study, and high distribution on the lungs in *in vivo* study.

## 1. Introduction

Coronavirus can cause multiple system infections including respiratory, digestive, and neurological systems in humans and other mammals [1]. The novel variant SARS-CoV-2 belongs to the subfamily of beta coronavirus. This makes the new virus the third zoonotic human coronavirus identified in this century. The last two zoonotic human coronaviruses which wreaked havoc in the global health system in the last two decades were the severe acute respiratory syndrome coronavirus (SARS-CoV) and Middle East respiratory syndrome coronavirus (MERS-CoV) [2]. The mortality rates were 10% for SARS-CoV and 37% for MERS-CoV [3]. The most up-to-date reports from the World Health Organization (WHO) showed that the SARS-CoV-2 epidemic has infected 13,876,441 people and claimed 593,087 lives worldwide at the time of writing [4]. According to the Chinese Guideline for Diagnosis and Treatment of SARS-CoV-2 Infection (Trial version 4), in most of the cases, the common symptoms are fever, drowsiness, and dry cough. In severe cases, serious symptoms may rapidly emerge, including acute respiratory distress syndrome, septic shock, metabolic acidosis, and coagulopathy. The susceptive groups are young children and elderly people [5]. Since the beginning of this epidemic, researchers have focused on new medications that could show potential to contain the transmission of the new virus and management of its collateral symptoms. The 4^th^ edition guidelines included the treatment including Chinese herbal medicine with 3 formulas to target 3 different stages of this disease. The symptoms in the initial stage of this disease are mild and relatively easy to manage compared to the severe stage. Herein, we aimed to investigate the modified herbal formula (Magnificent Atractylodes Rhizome powder; 神术散) designated for the management of the initial stage of this disease, namely, the stage of *cold dampness stagnation in the lung*, in Chinese medicine. It contains 9 herbs including *Atractylodes lancea (Thunb.) Dc.* (Atractylodes Rhizome; Cangzhu), *Citrus reticulata Blanco* (dried tangerine peel; Chenpi), *Magnolia officinalis Rehd. et Wils.* (Officinal Magnolia Bark; Houpo), *Agastache rugosa* (Agastaches Herba; Huoxiang), *Amomum tsaoko Crevost et Lemarie* (tsaoko fruit; Caoguo), *Ephedra sinica Stapf* (ephedra; Mahuang), *Notopterygium franchetii H. de Boiss.* (Incised Notopterygium Rhizome or Root; Qianghuo), *Zingiber officinale Roscoe* (fresh ginger; Shengjiang), and *Areca catechu* L. (areca seed; Binglang). The dosages of the ingredients are 15 grams for *Atractylodes lancea (Thunb.) Dc*., 6 grams for both *Amomum tsaoko Crevost et Lemarie* and *Ephedra sinica Stapf*, and 10 grams for the rest of ingredients [5]. However, scientific evidence is presently lacking to justify the claim of its effectiveness for the management of this disease.

The angiotensin-converting enzyme 2 (ACE2) receptor is viewed as the key protein in humans for the development of SARS-CoV-induced lung injury [6]. Since SARS-CoV-2 may target the same receptor to induce lung injury, it is proposed that computational molecular docking analysis is a feasible and rapid strategy to apply for the analysis of the interaction mechanism between the virus' spike glycoprotein and ACE2 receptor. However, there is presently no experimentally obtained structural model of the SARS-CoV-2 spike glycoprotein deposited yet in the Protein Databank (PDB) (http://www.rcsb.org). Despite several published and ongoing studies performed using docking analysis for the virus' proteins and ligands, the protein models applied in previous studies are based on the protein models from the SARS-CoV virus. However, the genetic data of the new viruses are available from GenBank (https://www.ncbi.nlm.nih.gov/genbank/). Therefore, in this study, we have modelled the spike glycoprotein of SARS-CoV-2 to examine the difference between SARS-CoV-2 and SARS-CoV and, in particular, to provide a molecular-level understanding of the difference in transmissibility and pathogenicity of SARS-CoV-2 compared to SARS-CoV.

## 2. Materials and Methods

The binding sites of the binding complex of the spike glycoprotein of both viruses and ACE2 were identified. The chemical compounds from these 9 herbs were explored for their binding affinities with ACE2. Finally, the identified chemical compounds were screened for their pharmacokinetic properties including ADME and toxicity to find a therapeutic agent with good potential to be a pharmaceutical candidate.

### 2.1. Model of the Binding Complex of SARS-CoV-2 Spike Glycoprotein and Angiotensin-Converting Enzyme 2

The genetic information of SARS-CoV-2 spike glycoprotein in PubMed with accession number as YP 009724390.1.1 was extracted into a FASTA format file. This sequence was used as input data for homology modelling in SWISS-MODEL (https://swissmodel.expasy.org/). Among the results, Model 2 was selected because of the high values of Coverage, GMQE, and QMEAN. Further detailed information is presented in the Supplementary SWISS-MODEL building result file and structure assessment file. The ACE2 protein structure was extracted from the Protein Data Bank with the PDB ID 1R4L in the PDB format. The ClusPro online server (https://cluspro.bu.edu/queue.php) was applied to perform protein-protein docking for SARS-CoV-2 spike glycoprotein and ACE2. Model 0 was selected out of the top 10 models in the balanced order (Supplementary ClusPro protein-protein docking file). The complex structural model was created and visualised using PyMOL Molecular Graphics System, Version 1.2r3pre, Schrödinger, LLC., with various colours applied to label the different chains of these two proteins to facilitate visualization and interpretation.

### 2.2. Model of the Binding Complex of SARS-CoV Spike Glycoprotein and Angiotensin-Converting Enzyme 2

The binding complex of these two proteins was extracted from Protein Data Bank with PDB ID 6ACG (https://www.rcsb.org/structure/6ACG) in PDB format. The structural model was created and visualised using PyMOL with various colours applied to label the different chains of these proteins to facilitate interpretation.

### 2.3. Identification of Binding Chains and Binding Sites of the SARS-CoV-2/SARS-CoV Spike Glycoproteins and Angiotensin-Converting Enzyme 2

PDBePISA (https://www.ebi.ac.uk/pdbe/pisa/) was employed for the identification of binding chains and binding sites of the SARS-CoV-2/SARS-CoV spike glycoproteins and ACE2. The interface result and hydrogen bonds are summarized in Table 1. The binding chains for the interactions of these two proteins were acquired from the interface result, while the binding sites were obtained from the results of the hydrogen bond analysis. Further detailed information is presented in the supplementary files with the file names as follows: SARS-CoV-2 and ACE2 interface results, SARS-CoV-2 chain B and ACE2 (chain D) binding sites result, SARS-CoV-2 chain C and ACE2 (chain D) binding sites result, SARS-CoV and ACE2 interface results, and SARS-CoV chain C and ACE2 (chain D) binding sites results. The above-identified binding chains and binding sites from these two viruses were compared to elucidate the similarity and difference.

### 2.4. Identification of Chemical Compounds from Designated Herbs

The TCMSP server (http://tcmspw.com/tcmsp.php) is an open online database with a large number of herbal entries with ADME properties, providing phytochemical information [7]. The Chinese character names of the identified herbs were used as input data for the search (Table 2). The chemical compound results of each herb were screened by the designed selection criterion, namely, that the logP value is not more than 3. This is based on the theory that logP values of 2-3 had been recommended as the cutoff value for hydration, which has been established as a benchmark for the solubility of compounds [8]. The results were saved as PDB files via PubChem for further analysis. Chemical compounds without PubChem ID were extracted directly from the database. The chemical structures of the compounds with strong binding affinities (≥9 kcal/mol) are summarized in Table 3 in Results. The chemical structures for the compounds with binding scores in the range of −7 kcal/mol to −9 kcal/mol are presented in the supplementary table.

### 2.5. Molecular Docking of the Chemical Compounds with Angiotensin-Converting Enzyme 2

The PyRx software was applied with AutoDock Vina for molecular docking. The binding affinity values are summarized in the spreadsheet in Excel file format (Supplementary docking result file). Molecular docking was performed using AutoDock Vina version 1.1.2 [9] (The Scripps Research Institute, La Jolla, CA, USA). The docking Graphical User Interface (GUI) frontend PyRx version 0.8 (https://pyrx.sourceforge.io/) (The Scripps Research Institute, La Jolla, CA, USA) was used to prepare all protein and ligand files for docking and for the generation of docking parameter input files. PyRx was employed to convert all protein and ligand PDB files into PDBQT format. Protonation states for titratable sidechains of the protein were based on those assigned using OpenBabel (OpenEye Scientific Software, Santa Fe, NM, USA) at pH 7. Gasteiger charges were applied to protein and ligands. Docking boxes were set using the “maximise” option in PyRx around the protein receptor in order to enable “blind” docking, in which the entire protein surface and accessible interior pockets were made available for potential binding of ligands. All dockings were performed with the default exhaustiveness value of 8. The dockings were semirigid, with full torsional flexibility allowed for the ligands, while the protein receptor structures were kept fixed. The cutoff value used to define strong binding affinity was set to be equal to or more than 7.0 kcal/mol. Therefore, the compounds with binding affinity values higher than this value were excluded for further study.

### 2.6. Text Mining for the Antiviral Activity of Identified Chemical Compounds

The identified chemical compounds with binding affinity (≥7.0 kcal/mol) were searched in PubChem for antiviral activity evidence from bioassay results. The chemical compounds with active results from bioassay studies against respiratory infection virus that have similar symptoms with COVID-19 were summarized with the minimal concentrations, study types, and references provided in Table 4.

### 2.7. Pharmacokinetic Property Screening and Prediction

The identified chemical compounds in PDB format were translated into MOL files using ChemDraw 3D version. SwissADME (http://www.swissadme.ch/) and PreADME (https://preadmet.bmdrc.kr/) were applied to predict pharmacokinetic properties including absorption, distribution, metabolism, and excretion (ADME) and toxicity. The results are summarized in a supplementary screening and prediction of ADME and toxicity table in terms of water solubility, Pharmacokinetic, Druglikeness, Medicinal Chemistry, Toxicity, and Eligibility. The screening criteria dictate that the chemical compound must be water-soluble, have high gastrointestinal absorption, satisfy Lipinski rule, and have low hERG inhibition risk (namely, hERG gene inhibition by chemical substance usually associated with the occurrence of prolonged QT syndrome, used as a standardised test for toxicity screening) [5].

### 2.8. Structural Analysis of the Identified Chemical Agent with Angiotensin-Converting Enzyme 2

The identified chemical compound and the ACE2 target were visualised in PyMOL to facilitate identification of specific residue interactions with active binding sites on the target. The PDBQT files of the binding ligand (chemical compound) and ACE2 obtained from AutoDock Vina were used as input files in PyMOL. The binding sites were highlighted in different colours and labelled with residue names.

## 3. Results

### 3.1. Model of the Binding Complex of SARS-CoV-2/SARS-CoV Spike Glycoproteins and Angiotensin-Converting Enzyme 2

**Figure 1:**
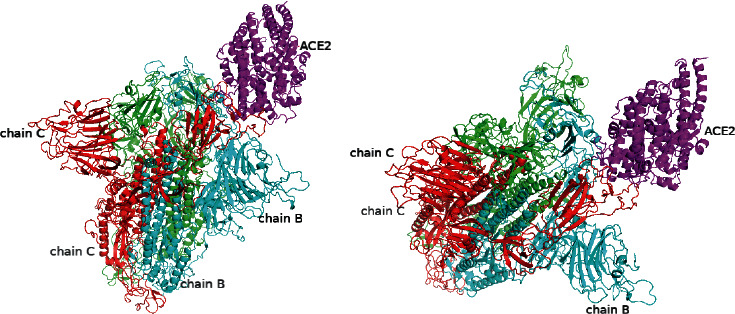
The simulation model of SARS-CoV-2 spike glycoprotein and angiotensin-converting enzyme 2.

The predicted model of the SARS-CoV-2 spike glycoprotein and angiotensin-converting enzyme 2 illustrated the interactions of these two proteins.  Figure 1 shows the SARS-CoV-2 spike glycoprotein coloured by its 3 different chains, with green for chain A, cyan for chain B, and red for chain C. ACE2 is coloured in magenta.  Figure 1 indicates that the chain B and chain C of SARS-CoV-2 spike glycoprotein both contact ACE2.

**Figure 2:**
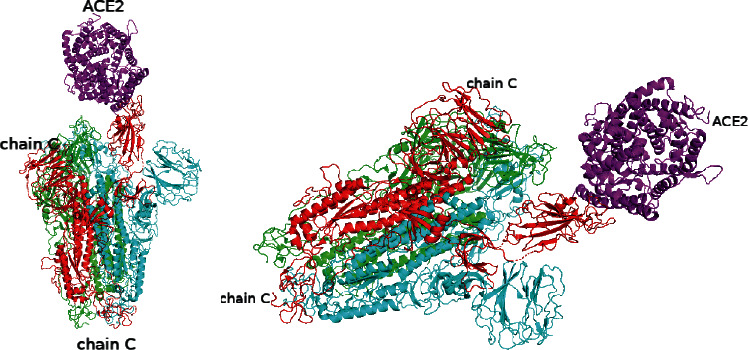
The simulation model of SARS-CoV spike glycoprotein and angiotensin-converting enzyme 2.

For comparison, the predicted model of the SARS-CoV spike glycoprotein and angiotensin-converting enzyme 2 illustrating the interactions of these two proteins is shown in Figure 2. The SARS-CoV spike glycoprotein is coloured by its 3 different chains, with green for chain A, cyan for chain B, and red for chain C. ACE2 is coloured in magenta. Inspection of the complex structural model shown in Figure 2 indicates that, in contrast to that of the SARS-CoV-2 spike glycoprotein, only chain C of the SARS-CoV spike glycoprotein is predicted to bind with ACE2.

### 3.2. Identification of the Binding Chains and Binding Site Residues of the SARS-CoV-2 Spike Glycoprotein and Angiotensin-Converting Enzyme 2

The interface result of the PDBePISA analysis for the binding complex of SARS-CoV-2 spike glycoprotein and ACE2 confirmed that both the chain B and chain C of SARS-CoV-2 spike glycoprotein form binding contacts with ACE2 (chain D). Detail information regarding the interaction between each of the relevant protein chains, interface contact areas, and estimated free energies of interactions is listed in Table 1. These results suggest that chain B and chain C of the SARS-CoV-2 spike glycoprotein contribute to the interaction with ACE2, with an estimated binding ΔG of −11.2 kcal/mol and an estimated ΔG of −5.3 kcal/mol, respectively.

The hydrogen bonds predicted to be formed between chain B of SARS-CoV-2 spike glycoprotein and ACE2 (chain D) showed that the main contributors to the interactions on chain B include THR333, ASN370, and ALA372. Likewise, the residues which make up binding sites on ACE2 included LYS600, SER254, and ALA614. The hydrogen bond-forming residues of chain C in SARS-CoV-2 spike glycoprotein include GLU484, GLN493, LYS417, ASN487, TYR489, GLN493, and TYR505. Likewise, the binding site residues on ACE2 which contribute to its interactions with SARS-CoV-2 chain C include ASP157, ASN159, ASP615, SER280, TYR252, and TYR613. Further detailed information regarding these key interactions is listed in Tables 5 and 6.

### 3.3. Identification of the Binding Chains and the Binding Site Residues of the SARS-CoV Spike Glycoprotein and Angiotensin-Converting Enzyme 2

The interface result of the PDBePISA analysis for the binding complex of the SARS-CoV spike glycoprotein and ACE2 confirmed that the chain C of SARS-CoV spike glycoprotein is the only chain which forms close contact with ACE2 (chain D), in contrast to SARS-CoV-2 in which both chains B and C form close contact. Further detailed information is listed in Table 7.

The hydrogen bond-forming residues of chain C in the SARS-CoV spike glycoprotein and ACE2 (chain D) complex showed that residues on chain C involved in binding ACE2 include ARG 426, TYR 436, ASN 473, TYR 475, THR 486, THR 487, ILE 489, TYR 484, and GLY 482. Likewise, the H-bond-forming binding site residues on ACE2 included GLN 24, GLN 42, ASP 38, TYR 41, TYR 83, GLN 325, ASN 330, and LYS 353. Further detailed information is listed in Table 8.

### 3.4. Comparison of the ACE2-Binding Regions of the SARS-CoV-2 and SARS-CoV Spike Glycoproteins

Comparison of the predicted binding chains and binding sites of the complexes demonstrated that SARS-CoV-2 had two chains (chain B and chain C) binding with ACE2, while in contrast, the SARS-CoV only had one chain (chain C) binding with ACE2. Examination of the specific residues involved in binding indicates that there is one common residue, at position 487, which is used by both SARS-CoV-2 and SARS-CoV spike glycoproteins to bind with ACE2.

### 3.5. Molecular Docking Screening of ACE2-Targeting Chemical Compounds from Designated Herbs

The chemical compounds from the herbs which satisfy the selection criteria amongst the nine herbs include the following: 11 chemical compounds from *Atractylodes lancea (Thunb.) Dc.* (Cangzhu), 31 chemical compounds from *Citrus reticulata Blanco* (Chenpi), 59 chemical compounds from *Magnolia officinalis Rehd. et Wils.* (Houpo), 38 chemical compounds from *Agastache rugosa* (Huoxiang), 30 chemical compounds from *Amomum tsaoko Crevost et Lemarie* (Caoguo), 204 chemical compounds from *Ephedra sinica Stapf* (Mahuang), 62 chemical compounds from *Notopterygium franchetii H. de Boiss*. (Qianghuo), 82 chemical compounds from *Zingiber officinale Roscoe* (Shengjiang), and 18 chemical compounds from *Areca catechu* L. (Binglang). Further detailed information is listed in Supplementary docking result file.

The binding affinity values for all docked compounds are presented in Supplementary docking result file. Chemical compounds which show binding affinity values greater than the cutoff value of 9 kcal/mol are atractylenolide III (9.1 kcal/mol) and oroxindin (9.5 kcal/mol) from *Atractylodes lancea (Thunb.) Dc.* (Cangzhu); hesperidin (10 kcal/mol) and naringin (10.5 kcal/mol) from *Citrus reticulata Blanco* (Chen pi); neohesperidin (10.6 kcal/mol) from *Magnolia officinalis Rehd. et Wils.* (Houpo); acanthoside B (9.1 kcal/mol), acteoside (9.5 kcal/mol), campneoside (9.3 kcal/mol), hyperin (10.2 kcal/mol), and orobanchoside (10.3 kcal/mol) from *Agastache rugosa* (Huoxiang); hirsutrin (10.1 kcal/mol), hyperin (10.2 kcal/mol), quercetin 3-o-glucoside (10.1 kcal/mol), quercetin 3-o-rhamnopyranosyl (9.7 kcal/mol), and quercetin 3-o-rutinoside (10.4 kcal/mol) from *Amomum tsaoko Crevost et Lemarie* (Caoguo); cosmetin (9.2 kcal/mol), hesperidin (10 kcal/mol), luteolin 7-O-glucuronide (9.3 kcal/mol), rutin (10.4 kcal/mol), tilianine (9.2 kcal/mol), and vitexin (9.0 kcal/mol) from *Ephedra sinica Stapf* (Mahuang); and chrysoeriol 7-rutinoside (10 kcal/mol), coumarin-glycoside (9.4 kcal/mol), and 6′-feruloylnodakenin (10.4 kcal/mol) from *Notopterygium franchetii H. de Boiss.* (Qianghuo). No compounds with satisfaction of the cutoff value from *Zingiber officinale Roscoe* (Shengjiang) and *Areca catechu* L. (Binglang) were identified. Table 3 demonstrates the results of chemical compounds and structures with high binding affinity scores (≥9 kcal/mol).

### 3.6. Text Mining Results for the Antiviral Activity of the Identified Chemical Compounds

From the findings of the *in vitro*, *in vivo*, and *in silico* studies, the chemical compounds with antirespiratory viral activities are apigenin, atractylenolide III, cosmetin, euxanthone, herbacetin, Hesperidin, hirsutrin, kaempferol, luteolin, quercetin, quercetin 3-o-rutinoside, and vitexin. The detailed information is presented in Table 4.

### 3.7. Pharmacokinetic Property Screening and Prediction

The chemical compound which satisfies the binding affinity and ADMET screening selection criteria is atractylenolide III from *Atractylodes lancea (Thunb.) Dc.* (Cangzhu) with eligible water solubility, high GI absorption, eligible druglikeness and low hERG inhibition risk. All the other chemical compounds were excluded due to unsuitable water solubility, GI absorption, druglikeness, and toxicity. Specifically, oroxindin was excluded with the reason of low GI absorption. For *Amomum tsaoko Crevost et Lemarie* (Caoguo), quercetin 3-o-glucoside, hirsutrin, and hyperin were excluded due to low GI absorption, violations of Lipinski's rules, and high hERG inhibition risk. Quercetin 3-o-rutinoside and quercetin 3-o-rhamnopyranosyl were excluded due to low GI absorption and violations of Lipinski's rules. For *Citrus reticulata Blanco* (Chenpi), hesperidin, hyperin, naringin, and orobanchoside were excluded due to low GI absorption, violations of Lipinski's rules, and high hERG inhibition risk. For *Magnolia officinalis Rehd. et Wils.*(Houpo), neohesperidin was excluded for the reasons of low GI absorption, violations of Lipinski's rules, and high hERG inhibition risk. For *Agastache rugosa* (Huoxiang), acanthoside B, acteoside, and campneoside were excluded due to low GI absorption and violations of Lipinski's rules. For *Notopterygium franchetii H. de Boiss* (Qianghuo), chrysoeriol 7-rutinoside was excluded due to low GI absorption, violations of Lipinski's rules, and high hERG inhibition risk. Coumarin- glycoside was excluded due to low GI absorption and medium hERG inhibition risk. 6′-Feruloylnodakenin was ruled out due to low GI absorption, violations of Lipinski's rules, and medium hERG inhibition risk. For *Ephedra sinica Stapf* (Mahuang), cosmetin was excluded due to low GI absorption and high hERG inhibition risk. Hesperidin and luteolin 7-O-glucuronide were excluded due to low GI absorption, violations of Lipinski's rules, and high hERG inhibition risk. Rutin was excluded due to low GI absorption and violations of Lipinski's rules. Tilianine was excluded due to low GI absorption and medium hERG inhibition risk. Vitexin was excluded due to low GI absorption and high hERG inhibition risk. Further detailed information of each identified chemical compounds' pharmacokinetic property screening and prediction is summarized in Table 9.

### 3.8. Structural Analysis of the Identified Chemical Agent with Angiotensin-Converting Enzyme 2

**Figure 3:**
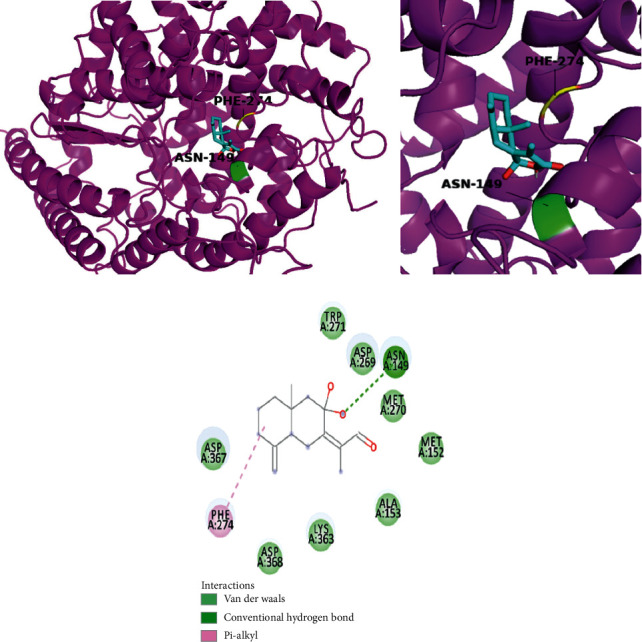
The simulation of atractylenolide III with ACE2 and 2-dimensional diagram.

The molecular graphic of the docking residue is shown in Figure 3, showing the predicted interaction between ACE2 and atractylenolide III, shown in ribbon form in magenta, and atractylenolide III, displayed as a “licorice” 3D structure. The key residues on ACE2 predicted to be involved in interaction with the ligand are also shown and involve a conventional hydrogen bond via ASN-149 (green) and pi-alkyl interaction via PHE 274 (yellow). As the binding results from hydrogen bond (H-bond) in chain B (Table 3) showed that SER254 can form a H-bond with SARS-CoV-2 spike glycoprotein. The binding results from hydrogen bond in chain C (Table 6) showed that ASP157, ASN159, TYR252 on ACE2 can form the H-bonds with SARS-CoV-2 spike glycoprotein. The ligand therefore binds in a region that shares a similar face as the ACE2 residues which are predicted to form contact with the SARS-CoV-2 spike glycoprotein. The ligand may therefore serve to disrupt, or weaken, ACE2-mediated virus-host cell interactions acting via this surface. A 2-dimensional diagram showed that the ASN 149 binding site in ACE2 is connected with a hydroxyl group in the molecule.

## 4. Discussion

The novel SARS-CoV-2 virus emerged to challenge the current medical system, exposing the shortfalls of existing pharmaceutical agents for its management. The Chinese Health Commission included Chinese herbal medicine amongst its current recommendations for disease management and have prescribed herbal formulas from its 4^th^ edition of SARS-CoV-2 virus management guidelines. In the initial stage, 9 herbs were prescribed for treating the symptoms, including chills, dry cough, dry throat, drowsiness, and chest tightness. Except *Amomum tsaoko Crevost et Lemarie* (Caoguo) and *Areca catechu* L. (Binglang), the other 7 herbs are among the high-frequency Chinese medicines for the management of pestilence throughout the history in China [19]. ACE2 receptors are viewed as the key protein in human for the development of SARS-CoV-induced lung injury [6]. Molecular docking was used to predict the binding mechanisms of both SARS-CoV-2 and SARS-CoV spike glycoproteins to ACE2, and it was identified that residue 487 for both viral proteins played a role in their binding to ACE2. The residue at position 487 for both SARS-CoV-2 and SARS-CoV spike glycoproteins has been proposed to be crucial for cross-species and human transmission for SARS-CoV [20]. Our results have echoed the findings from previous research that may strengthen the understanding of a similar role for the residue at this position of the new virus. Despite this similarity, there is still a large discrepancy between these two viruses in terms of binding chains and binding sites. These differences may be contributed by their genomic sequence diversity [20]. The binding results showed that SARS-CoV-2 could have two chains binding with ACE2 receptor rather than SARS-CoV with one chain binding with ACE2. More chain bindings or interactions and more energy consumptions indicate much stronger binding affinity for this new virus [21, 22]. The simulation predicted that chain C for both viruses is critical for the binding activities with ACE2. For SARS-CoV-2, the residues in chain C are LYS 417, GLU 484, ASN 487, TYR 489, GLN 493, and TYR 505. From the findings in another *in silico* study with the same PDB ID (6ACG) protein for modelling, the residue in position 505 (−4.23 kcal/mol) plays an important role for spike glycoprotein of SARS-CoV-2 in terms of the binding energy contribution in comparison with the residues in position 487 (−1.5 kcal/mol) and 489 (−3.0 kcal/mol) [22]. From another *in silico* study, the amino acid residues ASN157, ASN159, and SER 280 have contributed to the solvent at the surface of the ACE2 molecule (PDB ID : 1RIX) in the binding with SARS-CoV spike glycoproteins [23].

Molecular docking and pharmacokinetic screening were also used to identify atractylenolide III, from *Atractylodes lancea (Thunb.) Dc.* (Cangzhu) as a therapeutic agent with strong binding affinity with ACE2, which also satisfy selection criteria based on pharmacokinetic properties. The key predicted binding site residues on ACE2 are ASN 149, which form a conventional hydrogen bond by one hydroxyl group in the molecule and ASN 149 in ACE2. The results from *in vitro* inhibition assay showed atractylenolide III with antiviral activity instead of the same analog testing compounds atractylenolide I and atractylenolide II. Since the hydroxyl group is the structural difference between atractylenolide III and the same analog compounds, it can be deduced that the hydroxyl group may play a key role in the inhibition effect against porcine reproductive and respiratory syndrome virus [10]. The existing evidence could support the therapeutic potential of atractylenolide III for its anti-inflammatory activity, anti-porcine reproductive and respiratory syndrome virus activity, and heavy lung tissue distribution. Atractylenolide III (50 *µ*M and 100 *µ*M) possessed anti-inflammatory effects associated with the inhibition of nuclear factor-*κ*B (NF-*κ*B) and mitogen-activated protein kinases- (MAPK-) signaling pathways in lipopolysaccharide- (LPS-) induced RAW264.7 cells via suppression of the production of nitric oxide (NO), prostaglandin E2 (PGE2), tumour necrosis factor-alpha (TNF-*α*), and interleukin 6 (IL-6) [24]. Intriguingly, atractylenolide III has been shown to possess inhibitory effects against porcine reproductive and respiratory syndrome virus, which is of importance in the swine industry. This virus can cause similar symptoms of SARS-CoV-2 respiratory infection including fever, cough, and dyspnea. The 50% inhibited concentration (IC_50_) was 99.6 *µ*mol/L for this compound [10]. Atractylenolide III had been found to have high concentration level in the lung tissues of rats in pharmacokinetics and tissue distribution experiments [25]. It may shed light on the therapeutic potential of this compound for new virus-induced respiratory infections and inflammations. Despite the fact that our findings suggested other compounds with antirespiratory viral activity, the pharmacokinetic properties of these compounds are not eligible to be used as drug candidates. Therefore, atractylenolide III is the sole compound with antirespiratory activity and eligible pharmacokinetic properties to be considered as a drug candidate in our study. This finding is based on *in silico* study with limited evidence. It is noteworthy that there is no existing evidence from clinical studies to prove the efficacy of this formula. In order to acquire more solid evidential support, further *in vitro* and *in vivo* studies are required. The safety of Chinese herbal medicine is often a concern for its application and marketing. From the *Chinese Pharmacopoeia* 2015 edition, for the formula in the study, most of the herbs are safe to be applied but *Citrus reticulata Blanco* (dried tangerine peel; Chenpi), *Ephedra sinica Stapf* (ephedra; Mahuang), and *Areca catechu L.* (areca seed; Binglang) contain toxic substances. Aflatoxin can be found in both *Citrus reticulata Blanco* (dried tangerine peel; Chenpi) and *Areca catechu L.* (areca seed; Binglang). There are strict restrictions for aflatoxin in these herbs. For both *Citrus reticulata Blanco* (dried tangerine peel; Chenpi) and *Areca catechu L.* (areca seed; Binglang), Aflatoxin B1 cannot exceed 5 *µ*g per 1000 grams. Also, the total amount of aflatoxin G1, aflatoxin G2, aflatoxin B1, and aflatoxin B2 cannot be more than 10 *µ*g. The toxic compound ephedrine in *Ephedra sinica Stapf* (ephedra; Mahuang) may raise the biggest concerns among all the herbs in the formula. A series of toxic events and adverse effects had been reported that included arrhythmia, hepatotoxicity, cardiovascular toxicity, and dilated pupils after application of ephedra. Despite such safety concerns, the safe application of ephedra can be safeguarded by different processing techniques [26].

In conclusion, SARS-CoV-2 could bind with the ACE2 receptor at chain B and chain C to induce lung injuries in humans. The residue at position 487 may play a vital role as it did on SARS-CoV for the progression of lung injuries. Atractylenolide III is found to have a strong binding affinity with ACE2 by conventional hydrogen bond formation via ASN-149 and possess favourable pharmacokinetic properties, and it has been shown to exhibit anti-inflammatory effects and antiviral effects in a previous *in vitro* study and high distribution in the lungs in a previous *in vivo* study. All these findings support further research for the therapeutic effects of atractylenolide III for the management of this new virus.

## Figures and Tables

**Table 1:** PDBePISA interface result of SARS-CoV-2 spike glycoprotein and angiotensin-converting enzyme 2 (chain D).

Structure 1	Structure 2	Interface area (A^2^)	ΔiG (kcal/mol)	ΔiG (*P* value)
Chain B	Chain C	5285.6	−45.9	0.707
Chain A	Chain B	5263.4	−42.8	0.722
Chain A	Chain C	5248.1	−41.7	0.781
Chain D (ACE2)	Chain B	830.3	−11.2	0.341
Chain D (ACE2)	Chain C	712.7	−5.3	0.666

**Table 2:** The scientific names, pinyin names, and Chinese character names of the herbs.

No.	Scientific names	Pinyin names	Chinese character names in TCMSP
1	*Atractylodes lancea (Thunb.) Dc.*	Cangzhu	苍术
2	*Citrus reticulata Blanco*	Chenpi	陈皮
3	*Magnolia officinalis Rehd. et Wils.*	Houpo	厚朴
4	*Agastache rugosa*	Huoxiang	藿香
5	*Amomum tsaoko Crevost et Lemarie*	Caoguo	草果
6	*Ephedra sinica Stapf*	Mahuang	麻黄
7	*Notopterygium franchetii H. de Boiss.*	Qianghuo	羌活
8	*Zingiber officinale Roscoe*	Shengjiang	生姜
9	*Areca catechu* L.	Binglang	槟榔

**Table 3:** The results of bioactive compounds of the herbs with high binding affinity scores (≥9.0 kcal/mol).

Herb	Bioactive compounds	PubChem ID	Structure
*Atractylodes lancea (Thunb.) Dc.*	Atractylenolide III	155948	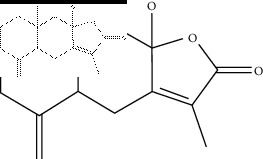
	Oroxindin	3084961	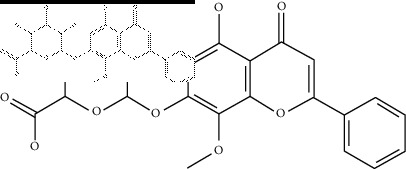
*Citrus reticulata Blanco*	Hesperidin	10621	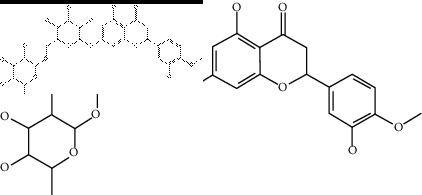
	Naringin	442428	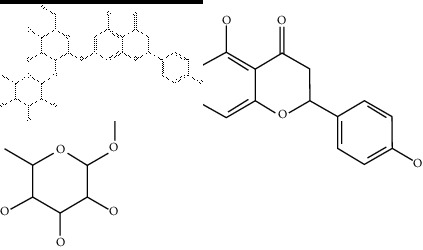
*Magnolia officinalis Rehd. et Wils.*	Neohesperidin	442439	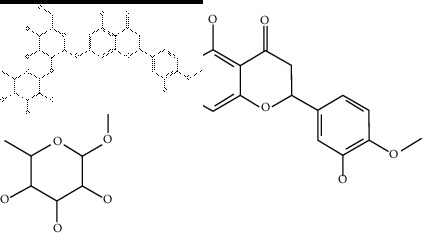
*Agastache rugosa*	Acanthoside B	443024	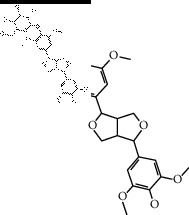
	Acteoside	5281800	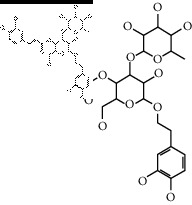
	Campneoside	5315651	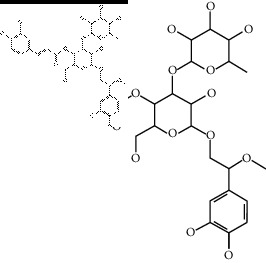
	Hyperin	5281643	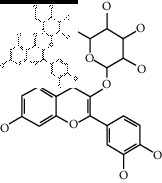
	Orobanchoside	6441894	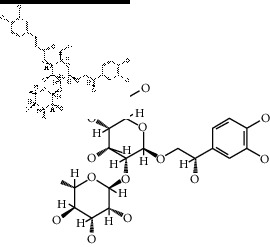
*Amomum tsaoko Crevost et Lemarie*	Hirsutrin	5280804	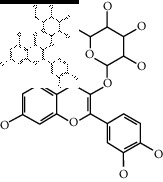
	Hyperin	5281643	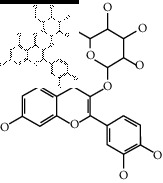
	Quercetin 3-o-glucoside	5280804	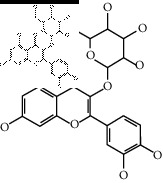
	Quercetin 3-o-rhamnopyranosyl	N/A	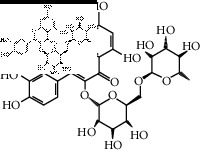
	Quercetin 3-o-rutinoside (synonymous: rutin)	5280805	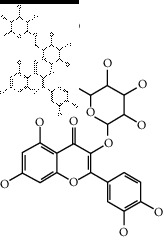
*Ephedra sinica Stapf*	Cosmetin	5280704	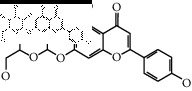
	Hesperidin	10621	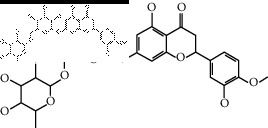
	Luteolin 7-O-glucuronide	5280601	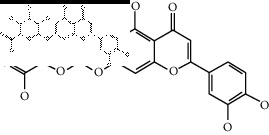
	Rutin	5280805	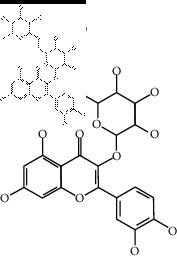
	Tilianine	5321954	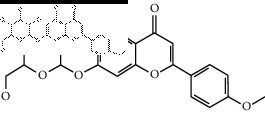
	Vitexin	5280441	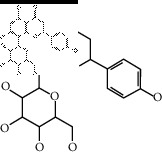
	Chrysoeriol 7-rutinoside	14374725	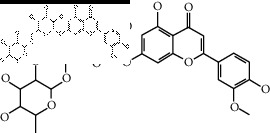
	Coumarin-glycoside	N/A	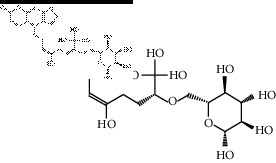
	6′-feruloylnodakenin	6439317	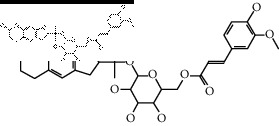

**Table 4:** The chemical compounds with active result from bioassay against respiratory infectious viral activity.

Herb	Chemical compound	Virus type	Minimal concentration	Study type	Reference
*Atractylodes lancea (Thunb.) Dc.*	Atractylenolide III	Porcine reproductive and respiratory syndrome virus	IC_50_ = 99.6 *µ*mol/L	*In vitro*	[10]
*Citrus reticulata Blanco*	Hesperidin	SARS-CoV-2	N/A	*In silico*	[11, 12]
*Agastache rugosa*	Quercetin	SARS-CoV	IC_50_ = 8.1 ± 0.3 *µ*m	*In vitro*	[13]
*Amomum tsaoko Crevost et Lemarie*					
*Ephedra sinica Stapf*					
*Agastache rugosa*	Quercetin	Influenza A virus H1N1 A/PR/8/34	EC_50_ = 43.1 *µ*m	*In vitro*	[14]
*Amomum tsaoko Crevost et Lemarie*					
*Ephedra sinica Stapf*					
*Agastache rugosa*	Quercetin	SARS-CoV	IC_50_ = 23.8 *µ*m	*In vitro*	[15]
*Amomum tsaoko Crevost et Lemarie*					
*Ephedra sinica Stapf*					
*Agastache rugosa*	Apigenin	Influenza A virus H1N1 A/PR/8/34	IC_50_ = 31.6 ± 0.9 *µ*m	*In vitro*	[16]
*Ephedra sinica Stapf*					
*Agastache rugosa*	Apigenin	Influenza A virus H3N2 A/Jinan/15/90	IC_50_ = 28.9 ± 0.7 *µ*m	*In vitro*	[16]
*Ephedra sinica Stapf*					
*Agastache rugosa*	Apigenin	Influenza A virus B/Jiangsu/10/2003	IC_50_ = 45.7 ± 2.3 *µ*m	*In vitro*	[16]
*Ephedra sinica Stapf*					
*Amomum tsaoko Crevost et Lemarie*	Quercetin, 3-o-rutinoside (Synonymous rutin)	Influenza A virus H1N1	IC_50_ = 34.4 ± 5.0 *µ*m	*In vitro*	[14]
*Ephedra sinica Stapf*					
*Amomum tsaoko Crevost et Lemarie*	Hirsutrin	Influenza A virus A/swine/OH/511445/2007 H1N1	ED_50_ = 1.2 *µ*m	*In vitro* and *in vivo*	[17]
*Ephedra sinica Stapf*	Cosmetin	Influenza A virus H1N1 A/PR/8/34	EC_50_ = 43.0 *µ*m	*In vitro*	[14]
*Ephedra sinica Stapf*	Vitexin	Influenza A virus H1N1 A/PR/8/34	IC_50_ = 46.5 ± 0.6 *µ*m	*In vitro*	[16]
*Ephedra sinica Stapf*	Vitexin	Influenza A virus H3N2 A/Jinan/15/90	IC_50_ = 45.1 ± 1.3 *µ*m	*In vitro*	[16]
*Ephedra sinica Stapf*	Vitexin	Influenza A virus B/Jiangsu/10/2003	IC_50_ = 49.6 ± 3.1 *µ*m	*In vitro*	[16]
*Ephedra sinica Stapf*	Luteolin	Influenza A virus H1N1 A/PR/8/34	IC_50_ = 33.7 ± 0.7 *µ*m	*In vitro*	[16]
*Ephedra sinica Stapf*	Luteolin	Influenza A virus H3N2 A/Jinan/15/90	IC_50_ = 32.6 ± 0.1 *µ*m	*In vitro*	[16]
*Ephedra sinica Stapf*	Luteolin	Influenza A virus B/Jiangsu/10/2003	IC_50_ = 53.3 ± 5.1 *µ*m	*In vitro*	[16]
*Ephedra sinica Stapf*	Herbacetin	Influenza A virus H1N1 A/PR/8/34	EC_50_ = 35.0 *µ*m	*In vitro*	[14]
*Ephedra sinica Stapf*	Kaempferol	Influenza A virus H1N1 A/PR/8/34	IC_50_ = 58.6 ± 0.6 *µ*m	*In vitro*	[16]
*Ephedra sinica Stapf*	Kaempferol	Influenza A virus H3N2 A/Jinan/15/90	IC_50_ = 38.1 ± 0.3 *µ*m	*In vitro*	[16]
*Ephedra sinica Stapf*	Kaempferol	Influenza A virus B/Jiangsu/10/2003	IC_50_ = 46.4 ± 0.8 *µ*m	*In vitro*	[16]
*Zingiber officinale Roscoe*	Euxanthone	Influenza A virus H1N1	IC_50_ = 23.54 ± 3.68 *µ*m	*In vitro*	[18]
*Zingiber officinale Roscoe*	Euxanthone	Influenza A virus H9N2	IC_50_ = 22.45 ± 3.45 *µ*m	*In vitro*	[18]
*Zingiber officinale Roscoe*	Euxanthone	Influenza A virus H1N1 swine	IC_50_ = 11.54 ± 0.35 *µ*m	*In vitro*	[18]
*Zingiber officinale Roscoe*	Euxanthone	Influenza A virus H1N1 (H274Y)	IC_50_ = 13.01 ± 0.41 *µ*m	*In vitro*	[18]

EC_50_: half maximal effective concentration; IC_50_: half maximal inhibitory concentration.

**Table 5:** Hydrogen bond-forming residues of chain B in SARS-CoV-2 spike glycoprotein and angiotensin-converting enzyme 2 (chain D).

##	Structure 1 (chain D)	Dist. (Å)	Structure 2 (chain B)
1	D: LYS 600[HZ2]	1.72	B: THR 333[OG1]
2	D: SER 254[O]	1.93	B: ASN 370[HD22]
3	D: ALA 614[O]	1.95	B: ALA 372[H]

**Table 6:** Hydrogen bond-forming residues of chain C in SARS-CoV-2 spike glycoprotein and angiotensin-converting enzyme 2 (chain D).

##	Structure 1 (chain D)	Dist. (Å)	Structure 2 (chain C)
1	D: ASP 157[H]	2.04	C: GLU 484[OE2]
2	D: ASN 159[HD22]	1.96	C: GLN 493[OE1]
3	D: ASP 615[OD1]	1.86	C: LYS 417[HZ1]
4	D: ASP 615[OD2]	1.75	C: LYS 417[HZ2]
5	D: SER 280[O]	2.05	C: ASN 487[HD22]
6	D: TYR 252[OH]	1.97	C: TYR 489[HH]
7	D: ASP 157[OD1]	2.18	C: GLN 493[HE22]
8	D: TYR 613[O]	1.91	C: TYR 505[HH]

**Table 7:** PDBePISA interface result of SARS-CoV spike glycoprotein and angiotensin-converting enzyme 2 (chain D).

Structure 1	Structure 2	Interface area (A^2^)	ΔiG (kcal/mol)	ΔiG (*P* value)
Chain A	Chain B	4679.7	−46.3	0.328
Chain A	Chain C	4326.6	−38.4	0.424
Chain B	Chain C	3749.1	−41.7	0.222
Chain C	Chain D (ACE2)	904.1	−8.8	0.325

**Table 8:** Hydrogen bond-forming residues of chain C in SARS-CoV spike glycoprotein and angiotensin-converting enzyme 2 (chain D).

##	Structure 1 (chain C)	Dist. (Å)	Structure 2 (chain D)
1	C: ARG 426[NH1]	2.69	D: GLN 325[OE1]
2	C: TYR 436[OH]	2.81	D: ASP 38[OD1]
3	C: TYR 436[OH]	2.72	D: ASP 38[OD2]
4	C: ASN 473[ND2]	3.46	D: GLN 24 [O]
5	C: ASN 473[ND2]	2.40	D: TYR 83[OH]
6	C: TYR 475[OH]	3.88	D: TYR 83[OH]
7	C: THR 486[OG1]	3.39	D: TYR 41[OH]
8	C: THR 487[N]	3.89	D: TYR 41[OH]
9	C: ILE 489[N]	3.65	D: GLN 325[OE1]
10	C: THR 486[O]	3.50	D: TYR 41[OH]
11	C: TYR 484[OH]	2.99	D: GLN 42[NE2]
12	C: TYR 436[OH]	2.77	D: GLN 42[NE2]
13	C: THR 486[O]	3.22	D: ASN 330[ND2]
14	C: GLY 482[O]	3.00	D: LYS 353[NZ]

**Table 9:** The screening and prediction of ADME and toxicity for the identified chemical compounds with antiviral activity (≥7 kcal/mol) from PreADME and SwissADME.

Herb	Chemical compound	Water solubility	Pharmacokinetics	Druglikeness	Toxicity	Eligibility
*Atractylodes lancea (Thunb.) Dc.*	Atractylenolide III	Log S (ESOL) −2.70 Solubility 4.93*e* − 01 mg/ml; 1.98*e* − 03 mol/l Class Soluble Log S (Ali) −2.71 Solubility 4.87*e* − 01 mg/ml; 1.96*e* − 03 mol/l Class Soluble Log S (SILICOS-IT) −3.15 Solubility 1.78*e* − 01 mg/ml; 7.16*e* − 04 mol/l Class Soluble	GI absorption High BBB permeant Yes P-gp substrate No CYP1A2 inhibitor No CYP2C19 inhibitor No CYP2C9 inhibitor No CYP2D6 inhibitor No CYP3A4 inhibitor No Log Kp (skin permeation) −6.32 cm/s	Lipinski Yes; 0 violation Ghose Yes Veber Yes Egan Yes Muegge Yes Bioavailability score 0.55	Algae at 0.0292313 Ames test mutagen Carcino Mouse negative Carcino Rat positive Daphnia at 0.0794231 hERG inhibition low risk Medaka at 0.00923153 Minnow at 0.00563542 TA100 10RLI negative TA100 NA negative TA1535 10RLI positive TA1535 NA negative	Yes

*Amomum tsaoko Crevost et Lemarie*	Hirsutrin	Log S (ESOL) −3.04 Solubility 4.23*e* − 01 mg/ml; 9.10*e* − 04 mol/l Class Soluble Log S (Ali) −4.35 Solubility 2.10*e* − 02 mg/ml; 4.51*e* − 05 mol/l Class Moderately soluble Log S (SILICOS-IT) −1.51 Solubility 1.43*e* + 01 mg/ml; 3.08*e* − 02 mol/l Class Soluble	GI absorption Low BBB permeant No P-gp substrate No CYP1A2 inhibitor No CYP2C19 inhibitor No CYP2C9 inhibitor No CYP2D6 inhibitor No CYP3A4 inhibitor No Log Kp (skin permeation) −8.88 cm/s	Lipinski No; 2 violations: NorO > 10, NHorOH > 5 Ghose No; 1 violation: WLOGP < -0.4 Veber No; 1 violation: TPSA > 140 Egan No; 1 violation: TPSA > 131.6 Muegge No; 3 violations: TPSA > 150, H-acc > 10, H-don > 5 Bioavailability score 0.17	Algae at 0.0220269 Ames test non-mutagen Carcino Mouse negative Carcino Rat negative Daphnia at 1.47843 hERG inhibition high risk Medaka at 3.73921 Minnow at 1.38214 TA100 10RLI negative TA100 NA negative TA153510RLI negative TA1535 NA negative	No
	Quercetin	Log S (ESOL) −3.16 Solubility 2.11*e* − 01 mg/ml; 6.98*e* − 04 mol/l Class Soluble Log S (Ali) −3.91 Solubility 3.74*e* − 02 mg/ml; 1.24*e* − 04 mol/l Class Soluble Log S (SILICOS-IT) −3.24 Solubility 1.73*e* − 01 mg/ml; 5.73*e* − 04 mol/l Class Soluble	GI absorption High BBB permeant No P-gp substrate No CYP1A2 inhibitor Yes CYP2C19 inhibitor No CYP2C9 inhibitor No CYP2D6 inhibitor Yes CYP3A4 inhibitor Yes Log Kp (skin permeation) −7.05 cm/s	Lipinski Yes; 0 violation Ghose Yes Veber Yes Egan Yes Muegge Yes Bioavailability score 0.55	Algae at 0.0378136 Ames test mutagen Carcino Mouse negative Carcino Rat positive Daphnia at 0.214345 hERG inhibition medium risk Medaka at 0.0778806 Minnow at 0.0335026 TA100 10RLI negative TA100 NA positive TA1535 10RLI negative TA1535 NA negative	No
	Quercetin, 3-o-glucoside (synonymous hirsutrin)	Log S (ESOL) −3.04 Solubility 4.23*e* − 01 mg/ml; 9.10*e* − 04 mol/l Class Soluble Log S (Ali) −4.35 Solubility 2.10*e* − 02 mg/ml; 4.51*e* − 05 mol/l Class Moderately soluble Log S (SILICOS-IT) −1.51 Solubility 1.43*e* + 01 mg/ml; 3.08*e* − 02 mol/l Class Soluble	GI absorption Low BBB permeant No P-gp substrate No CYP1A2 inhibitor No CYP2C19 inhibitor No CYP2C9 inhibitor No CYP2D6 inhibitor No CYP3A4 inhibitor No Log Kp (skin permeation) −8.88 cm/s	Lipinski No; 2 violations: NorO > 10, NHorOH > 5 Ghose No; 1 violation: WLOGP < −0.4 Veber No; 1 violation: TPSA > 140 Egan No; 1 violation: TPSA > 131.6 Muegge No; 3 violations: TPSA > 150, H-acc > 10, H-don > 5 Bioavailability score 0.17	Algae at 0.0220269 Ames test non-mutagen Carcino Mouse negative Carcino Rat negative Daphnia at 1.47843 hERG inhibition high risk Medaka at 3.73921 Minnow at 1.38214 TA100 10RLI negative TA100 NA negative TA1535 10RLI negative TA1535 NA negative	No
	Quercetin, 3-o-rutinoside Synonymous Rutin	Log S (ESOL) −3.30 Solubility 3.08*e* − 01 mg/ml; 5.05*e* − 04 mol/l Class Soluble Log S (Ali) −4.87 Solubility 8.30*e* − 03 mg/ml; 1.36*e* − 05 mol/l Class Moderately soluble Log S (SILICOS-IT) −0.29 Solubility 3.15*e* + 02 mg/ml; 5.15*e* − 01 mol/l Class Soluble	GI absorption Low BBB permeant No P-gp substrate Yes CYP1A2 inhibitor No CYP2C19 inhibitor No CYP2C9 inhibitor No CYP2D6 inhibitor No CYP3A4 inhibitor No Log Kp (skin permeation) −10.26 cm/s	Lipinski No; 3 violations: MW > 500, NorO > 10, NHorOH > 5 Ghose No; 4 violations: MW > 480, WLOGP < −0.4, MR > 130, #atoms > 70 Veber No; 1 violation: TPSA > 140 Egan No; 1 violation: TPSA > 131.6 Muegge No; 4 violations: MW > 600, TPSA > 150, H-acc > 10, H-don > 5 Bioavailability score 0.17	Algae at 0.0069585 Ames test non-mutagen Carcino Mouse negative Carcino Rat negative Daphnia at 2.55255 hERG inhibition ambiguous Medaka at 12.3433 Minnow at 5.4421 TA100 10RLI negative TA100 NA negative TA1535 10RLI negative TA1535 NA negative	No

*Citrus reticulata Blanco*	Apigenin	Log S (ESOL) −3.94 Solubility 3.07*e* − 02 mg/ml; 1.14*e* − 04 mol/l Class Soluble Log S (Ali) −4.59 Solubility 6.88*e* − 03 mg/ml; 2.55*e* − 05 mol/l Class Moderately soluble Log S (SILICOS-IT) −4.40 Solubility 1.07*e* − 02 mg/ml; 3.94*e* − 05 mol/l Class Moderately soluble	GI absorption High BBB permeant No P-gp substrate No CYP1A2 inhibitor Yes CYP2C19 inhibitor No CYP2C9 inhibitor No CYP2D6 inhibitor Yes CYP3A4 inhibitor Yes Log Kp (skin permeation) −5.80 cm/s	Lipinski Yes; 0 violation Ghose Yes Veber Yes Egan Yes Muegge Yes Bioavailability Score 0.55	Algae at 0.0527482 Ames test mutagen Carcino Mouse positive Carcino Rat positive Daphnia at 0.130131 hERG inhibition medium risk Medaka at 0.0280583 Minnow at 0.0152727 TA100 10RLI positive TA100 NA positive TA1535 10RLI negative TA1535 NA negative	No
	Hesperidin	Log S (ESOL) −3.28 Solubility 3.19*e* − 01 mg/ml; 5.23*e* − 04 mol/l Class Soluble Log S (Ali) −4.33 Solubility 2.88*e* − 02 mg/ml; 4.72*e* − 05 mol/l Class Moderately soluble Log S (SILICOS-IT) −0.58 Solubility 1.60*e* + 02 mg/ml; 2.62*e* − 01 mol/l Class Soluble	GI absorption Low BBB permeant No P-gp substrate Yes CYP1A2 inhibitor No CYP2C19 inhibitor No CYP2C9 inhibitor No CYP2D6 inhibitor No CYP3A4 inhibitor No Log Kp (skin permeation) −10.12 cm/s	Lipinski No; 3 violations: MW > 500, NorO > 10, NHorOH > 5 Ghose No; 4 violations: MW > 480, WLOGP < −0.4, MR > 130, #atoms > 70 Veber No; 1 violation: TPSA > 140 Egan No; 1 violation: TPSA > 131.6 Muegge No; 4 violations: MW > 600, TPSA > 150, H-acc > 10, H-don > 5 Bioavailability score 0.17	Algae at 0.00697422 Ames test non-mutagen Carcino Mouse negative Carcino Rat negative Daphnia at 0.961213 hERG inhibition high risk Medaka at 1.81708 Minnow at 1.91089 TA100 10RLI negative TA100 NA negative TA1535 10RLI negative TA1535 NA negative	No

*Ephedra sinica Stapf*	Cosmetin	Log S (ESOL) −3.78 Solubility 7.19*e* − 02 mg/ml; 1.66*e* − 04 mol/l Class Soluble Log S (Ali) −5.00 Solubility 4.32*e* − 03 mg/ml; 9.99*e* − 06 mol/l Class Moderately soluble Log S (SILICOS-IT) −2.69 Solubility 8.77*e* − 01 mg/ml; 2.03*e* − 03 mol/l Class Soluble	GI absorption Low BBB permeant No P-gp substrate Yes CYP1A2 inhibitor No CYP2C19 inhibitor No CYP2C9 inhibitor No CYP2D6 inhibitor No CYP3A4 inhibitor No Log Kp (skin permeation) −7.65 cm/s	Lipinski Yes; 1 violation: NHorOH > 5 Ghose Yes Veber No; 1 violation: TPSA > 140 Egan No; 1 violation: TPSA > 131.6 Muegge No; 2 violations: TPSA > 150, H-don > 5 Bioavailability Score 0.55	Algae at 0.0230381 Ames test mutagen Carcino Mouse positive Carcino Rat negative Daphnia at 0.5109 hERG inhibition high risk Medaka at 0.46598 Minnow at 0.447713 TA100 10RLI negative TA100 NA negative TA1535 10RLI negative TA1535 NA negative	No
	Herbacetin	Log S (ESOL) −3.55 Solubility 8.46*e* − 02 mg/ml; 2.80*e* − 04 mol/l Class Soluble Log S (Ali) −4.56 Solubility 8.29*e* − 03 mg/ml; 2.74*e* − 05 mol/l Class Moderately soluble Log S (SILICOS-IT) −3.24 Solubility 1.73*e* − 01 mg/ml; 5.73*e* − 04 mol/l Class Soluble	GI absorption High BBB permeant No P-gp substrate No CYP1A2 inhibitor Yes CYP2C19 inhibitor No CYP2C9 inhibitor No CYP2D6 inhibitor Yes CYP3A4 inhibitor Yes Log Kp (skin permeation) −6.60 cm/s	Lipinski Yes; 0 violation Ghose Yes Veber Yes Egan Yes Muegge Yes Bioavailability score 0.55	Algae at 0.0368839 Ames test mutagen Carcino Mouse negative Carcino Rat positive Daphnia at 0.206834 hERG inhibition medium risk Medaka at 0.0728344 Minnow at 0.0352894 TA100 10RLI negative TA100 NA positive TA1535 10RLI negative TA1535 NA negative	No
	Hesperidin (duplicated)	Log S (ESOL) −3.28 Solubility 3.19*e* − 01 mg/ml; 5.23*e* − 04 mol/l Class Soluble Log S (Ali) −4.33 Solubility 2.88*e* − 02 mg/ml; 4.72*e* − 05 mol/l Log S (SILICOS-IT) −0.58 Solubility 1.60*e* + 02 mg/ml; 2.62*e* − 01 mol/l Class Soluble	GI absorption Low BBB permeant No P-gp substrate Yes CYP1A2 inhibitor No CYP2C19 inhibitor No CYP2C9 inhibitor No CYP2D6 inhibitor No CYP3A4 inhibitor No Log Kp (skin permeation) −10.12 cm/s	Lipinski No; 3 violations: MW > 500, NorO > 10, NHorOH > 5 Ghose No; 4 violations: MW > 480, WLOGP < −0.4, MR > 130, #atoms > 70 Veber No; 1 violation: TPSA > 140 Egan No; 1 violation: TPSA > 131.6 Muegge No; 4 violations: MW > 600, TPSA > 150, H-acc > 10, H-don > 5 Bioavailability score 0.17	Algae at 0.00697422 Ames test non-mutagen Carcino Mouse negative Carcino Rat negative Daphnia at 0.961213 hERG inhibition high risk Medaka at 1.81708 Minnow at 1.91089 TA100 10RLI negative TA100 NA negative TA1535 10RLI negative TA1535 NA negative	No
	Kaempferol	Log S (ESOL) −3.31 Solubility 1.40*e* − 01 mg/ml; 4.90*e* − 04 mol/l Class Soluble Log S (Ali) −3.86 Solubility 3.98*e* − 02 mg/ml; 1.39*e* − 04 mol/l Class Soluble Log S (SILICOS-IT) −3.82 Solubility 4.29*e* − 02 mg/ml; 1.50*e* − 04 mol/l Class Soluble	GI absorption High BBB permeant No P-gp substrate No CYP1A2 inhibitor Yes CYP2C19 inhibitor No CYP2C9 inhibitor No CYP2D6 inhibitor Yes CYP3A4 inhibitor Yes Log Kp (skin permeation) −6.70 cm/s	Lipinski Yes; 0 violation Ghose Yes Veber Yes Egan Yes Muegge Yes Bioavailability score 0.55	Algae at 0.0483223 Ames test mutagen Carcino Mouse negative Carcino Rat positive Daphnia at 0.196882 hERG inhibition medium risk Medaka at 0.0642539 Minnow at 0.0294885 TA100 10RLI negative TA100 NA positive TA1535 10RLI negative TA1535 NA negative	No
	Luteolin	Log S (ESOL) −3.71 Solubility 5.63*e* − 02 mg/ml; 1.97*e* − 04 mol/l Class Soluble Log S (Ali) −4.51 Solubility 8.84*e* − 03 mg/ml; 3.09*e* − 05 mol/l Class Moderately soluble Log S (SILICOS-IT) −3.82 Solubility 4.29*e* − 02 mg/ml; 1.50*e* − 04 mol/l Class Soluble	GI absorption High BBB permeant No P-gp substrate No CYP1A2 inhibitor Yes CYP2C19 inhibitor No CYP2C9 inhibitor No CYP2D6 inhibitor Yes CYP3A4 inhibitor Yes Log Kp (skin permeation) −6.25 cm/s	Lipinski Yes; 0 violation Ghose Yes Veber Yes Egan Yes Muegge Yes Bioavailability score 0.55	Algae at 0.0416314 Ames test mutagen Carcino Mouse negative Carcino Rat positive Daphnia at 0.139325 hERG inhibition medium risk Medaka at 0.0329883 Minnow at 0.0169052 TA100 10RLI negative TA100 NA positive TA1535 10RLI negative TA1535 NA negative	No
	Rutin (duplicated)	Log S (ESOL) −3.30 Solubility 3.08*e* − 01 mg/ml; 5.05*e* − 04 mol/l Class Soluble Log S (Ali) −4.87 Solubility 8.30*e* − 03 mg/ml; 1.36*e* − 05 mol/l Class Moderately soluble Log S (SILICOS-IT) −0.29 Solubility 3.15*e* + 02 mg/ml; 5.15*e* − 01 mol/l Class Soluble	GI absorption Low BBB permeant No P-gp substrate Yes CYP1A2 inhibitor No CYP2C19 inhibitor No CYP2C9 inhibitor No CYP2D6 inhibitor No CYP3A4 inhibitor No Log Kp (skin permeation) −10.26 cm/s	Lipinski No; 3 violations: MW > 500, NorO > 10, NHorOH > 5 Ghose No; 4 violations: MW > 480, WLOGP < −0.4, MR > 130, #atoms > 70 Veber No; 1 violation: TPSA > 140 Egan No; 1 violation: TPSA > 131.6 Muegge No; 4 violations: MW > 600, TPSA > 150, H-acc > 10, H-don > 5 Bioavailability score 0.17	Algae at 0.0069585 Ames test non-mutagen Carcino Mouse negative Carcino Rat negative Daphnia at 2.55255 hERG inhibition ambiguous Medaka at 12.3433 Minnow at 5.4421 TA100 10RLI negative TA100 NA negative TA1535 10RLI negative TA1535 NA negative	No
	Vitexin	Log S (ESOL) −2.84 Solubility 6.29*e* − 01 mg/ml; 1.46*e* − 03 mol/l Class soluble Log S (Ali) −3.57 Solubility 1.16*e* − 01 mg/ml; 2.68*e* − 04 mol/l Class soluble Log S (SILICOS-IT) −2.38 Solubility 1.81*e* + 00 mg/ml; 4.20*e* − 03 mol/l Class Soluble	GI absorption Low BBB permeant No P-gp substrate No CYP1A2 inhibitor No CYP2C19 inhibitor No CYP2C9 inhibitor No CYP2D6 inhibitor No CYP3A4 inhibitor No Log Kp (skin permeation) −8.79 cm/s	Lipinski Yes; 1 violation: NHorOH > 5 Ghose Yes Veber No; 1 violation: TPSA > 140 Egan No; 1 violation: TPSA > 131.6 Muegge No; 2 violations: TPSA > 150, H-don > 5 Bioavailability score 0.55	Algae at 0.0287951 Ames test non-mutagen Carcino Mouse positive Carcino Rat negative Daphnia at 0.775983 hERG inhibition high risk Medaka at 1.05813 Minnow at 0.763184 TA100 10RLI negative TA100 NA negative TA1535 10RLI negative TA1535 NA negative	No

*Zingiber officinale Roscoe*	Euxanthone	Log S (ESOL) −3.63 Solubility 5.37*e* − 02 mg/ml; 2.35*e* − 04 mol/l Class Soluble Log S (Ali) −3.94 solubility 2.62*e* − 02 mg/ml; 1.15*e* − 04 mol/l Class Soluble Log S (SILICOS-IT) −4.14 Solubility 1.64*e* − 02 mg/ml; 7.18*e* − 05 mol/l Class Moderately soluble	GI absorption High BBB permeant Yes P-gp substrate No CYP1A2 inhibitor Yes CYP2C19 inhibitor No CYP2C9 inhibitor No CYP2D6 inhibitor Yes CYP3A4 inhibitor Yes Log Kp (skin permeation) −5.70 cm/s	Lipinski Yes; 0 violation Ghose Yes Veber Yes Egan Yes Muegge Yes Bioavailability score 0.55	Algae at 0.0653294 Ames test mutagen Carcino Mouse negative Carcino Rat positive Daphnia at 0.142765 hERG inhibition medium risk Medaka at 0.0311152 Minnow at 0.0148244 TA100 10RLI positive TA100 NA positive TA1535 10RLI positive TA1535 NA negative	No

## Data Availability

The data are available upon request to the corresponding author.

## Abbreviations

ACE2: Angiotensin-converting enzyme 2
ADME: Absorption, distribution, metabolism, and excretion
H-bond: Hydrogen bond
IL-6: Interleukin 6
LPS: Lipopolysaccharide
MAPK: Mitogen-activated protein kinases
MERS-CoV: Middle East respiratory syndrome coronavirus
NF-*κ*B: Nuclear factor-*κ*B
NO: Nitric oxide
PGE2: Prostaglandin E2
SARS-Cov: Severe acute respiratory syndrome coronavirus
TNF-*α*: Tumour necrosis factor-alpha
WHO: World Health Organization.
