# Supplementary figures and images for: Screening for a Potential Therapeutic Agent from the Herbal Formula in the 4th Edition of the Chinese National Guidelines for the Initial-Stage Management of COVID-19 via Molecular Docking
Source: Evid Based Complement Alternat Med. 2020 Dec 22;2020:3219840. doi: 10.1155/2020/3219840 (PMC7759025; doi:10.1155/2020/3219840)

## COVID-19

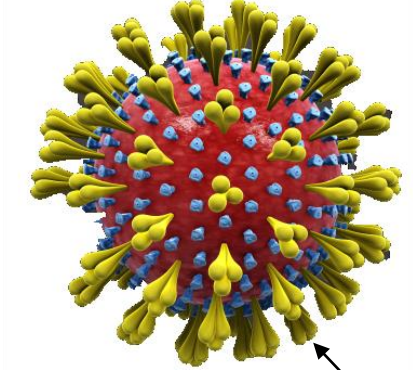

Spike Glycoprotein

## Spike Glycoprotein binding ACE2

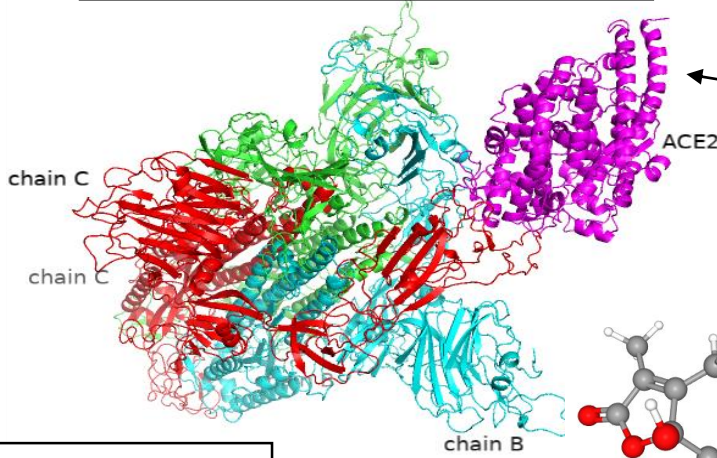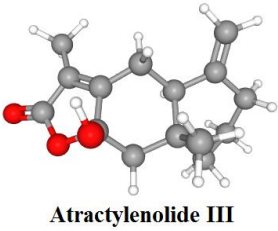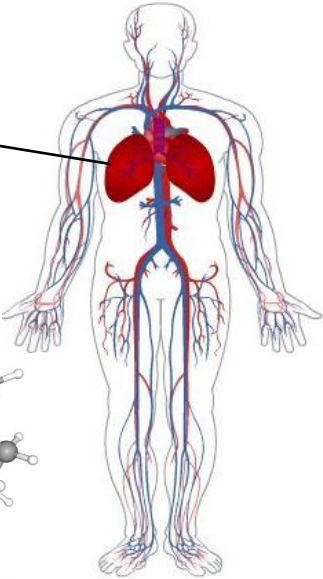

Supplement: Supplementary Materials — The supplementary files and tables are available in the separate files. [file 3219840.f1.pdf]
